# Taking care of people experiencing homelessness: a community case study on the practice of the Volunteer Association “A doctor for you” in Brescia, Italy

**DOI:** 10.3389/fpubh.2025.1611410

**Published:** 2025-08-26

**Authors:** A. Pasini, E. Pasini, F. Puccio, C. Seddio, P. Bocci, R. Lovati, M. Amadini

**Affiliations:** ^1^Department of Pedagogy/Education, Research Centre on Family and Childhood Education, Catholic University of Sacred Heart, Brescia, Italy; ^2^Department of Clinical and Experimental Sciences, University of Brescia, Brescia, Italy; ^3^Department of Humanity, Ruaha Catholic University, Iringa, Tanzania; ^4^Volunteer Association ‘Un Medico x Te', Brescia, Italy

**Keywords:** caring approaches, health, right to health, invisibility, multi-perspective approaches

## Abstract

**Introduction:**

Although homelessness is a much-studied phenomenon today, statistics indicate a steady increase in homelessness globally. UN General Assembly itself recommends implementing strategies through the commitment of the various stakeholders involved, among which health services are at the forefront. This current community case study details a specific research-practice partnership strategy—led in Brescia (Italy) in 2023 by the Association of volunteer doctors “Un Medico x Te” (A doctor for you) and the Research Center on Family and Childhood Education (CESPEFI) of the Università Cattolica del Sacro Cuore—as an attempt to deepen the evaluation of peoples experiencing homelessness' health conditions in Brescia urban area and to promote best practices in granting population experiencing homelessness sustainable health-care approaches.

**Method:**

In phase 1, since chronic liver diseases are very common in people experiencing homelessness, to investigate the presence of these pathologies, the Association “Un Medico x Te” carried out a preliminary observational retrospective echocardiographic cohort study in the population experiencing homelessness hosted in Brescia's housing services. In phase 2, CESPEFI led a research project to investigate the way health and healthcare is conceived by the homeless population living in Brescia urban area, according to the ethnographic methodological framework. Qualitative data were coded to explore emerging themes concerning the best practices to approach the population experiencing homelessness and to grant them appropriate care. At the end of the process internal guidelines were drawn up, based on greater attention to the relationship between doctors and patients experiencing homelessness and to the sustainability of the care-process.

**Results:**

Chronic hepatic diseases were more common and severe in people experiencing homelessness than general population and it was related to the duration of the homeless condition. Taking care of the particular aspects of the relationships between doctors and patients experiencing homelessness made clinical research and health care more effective.

**Discussion:**

This study demonstrates that a multi-disciplinary approach is strategic in order to respond to such a complex phenomenon as homelessness is; not-adopting a multidisciplinary approach may itself represent an additional mechanism of exclusion. Further studies are needed to explore innovative strategies to better face the problem of how: 1) to approach patients experiencing homelessness and 2) to implement appropriate medical assistance.

## 1 Introduction

Homelessness, characterized by the convergence of housing deprivation and social hardship within a dynamic, processual framework, increasingly demands multidisciplinary and intersectional analyses. This complex phenomenon is defined by a multiplicity of marginalizing factors (1), encompassing both material and immaterial dimensions.

Material marginalization includes lack of adequate housing, employment, economic resources, and access to healthcare. Immaterial marginalization comprises deficiencies in familial and social support networks, and the absence of professional connections. Collectively, these factors constitute a severe form of social exclusion (2, 3).

Empirical evidence from the lives of individuals experiencing homelessness indicates that their complex social circumstances are frequently correlated with other critical issues, such as physical and mental health disorders, substance dependencies, and involvement with the criminal justice system (4). Multidisciplinary and integrated approaches are therefore essential for comprehensive analysis and effective intervention. Furthermore, professional experience among social workers suggests that interdisciplinary collaboration and interconnected service delivery enhance engagement with the population experiencing homelessness (5).

Despite extensive research and the identification of numerous valuable intervention methodologies (6–8), the overarching goal of eliminating homelessness remains elusive, with global statistics indicating a persistent upward trend. While an internationally standardized definition of homelessness is lacking, and statistical definitions vary considerably across nations (9), data indicates that over two million individuals within OECD (Organization for Economic Cooperation and Development) countries experience homelessness. Since 2022, following the COVID-19 pandemic, most countries with available data have reported an increase in the number of people experiencing homelessness. For example, in Ireland, the Netherlands, the United Kingdom (England), and the United States, the number of people experiencing homelessness increased by more than 10 index points between 2022 and 2023 (10).

Globally, in 2024 the United Nations Human Settlements Programme (UN-Habitat) estimates that between 1.6 billion and 3 billion people lack adequate housing. Over 1.12 billion people lived in informal settlements and slums in 2022, 130 million more than in 2015. At least 330 million experience absolute homelessness, according to the Institute of Global Homelessness. Millions more face rising housing costs, unaffordable rents, evictions, energy poverty and unsafe living conditions, worsened by climate change (11).

Faced with this persistent challenge, the UN General Assembly (12) advocates for implementing strategies through the collaborative commitment of various stakeholders, with health services playing a central role.

Indeed, data shows population experiencing homelessness have a mortality rate eight to 12 times higher for men and women respectively than the average population (13), and they have elevated prevalence of cardiovascular, pulmonary and hepatic chronic diseases with significant social and economic impact (14).

Consequently, addressing the healthcare needs of individuals experiencing homelessness and evaluating their health status is ethically fundamental to informing public health and social policy (15).

In light of the previous considerations, traditional approaches to healthcare, encompassing both quality of life and conventional medical interventions, appear to be sub-optimal for addressing the needs of people experiencing homelessness. This necessitates the development of innovative methods of study and intervention tailored to the population experiencing homelessness. Indeed, an increasing body of research from various countries recommends the adoption of holistic, systemic strategies and navigation services to engage “homeless services users” and to enhance both community and healthcare resources, thereby facilitating the fulfillment of healthcare needs” (16–18).

The present study therefore aimed to deepen understanding of the living conditions of individuals experiencing homelessness in Brescia urban area, considering both their physical health and social functioning. In particular, it sought to analyse the most effective approaches for engaging with this population, with the goal of developing—at least within the organization Un Medico × Te—improved communication, relational, and treatment strategies, thereby better safeguarding the right to health for individuals who often lack full or partial access to healthcare services.

## 2 Context

In this regard, we describe the experience of the Association of volunteer doctors “Un Medico x Te” (A doctor for you), which works in Brescia (Northern Italy) in favor of the city's population experiencing homelessness.

Brescia urban area has a considerable presence of people experiencing homelessness, probably also because of its significant number of services for severe adult marginality.

The Statistics and Data Study Center of the Municipal Registry Office of the City of Brescia estimates the resident population in 2023 at 200,691 citizens, of whom 552 are experiencing homelessness—however, this figure only includes the generality of citizens with a fictitious residence in via S. Marie del Mare, and therefore does not take into account the additional presence of other people in the area who are known to be experiencing homelessness in reality (perhaps even known to low-threshold services), but who are not known to be registered (19).

In November 2017, the Municipality of Brescia constituted the “Cabina di regia” (control room) and the “Tavolo tecnico” (technical working group) on severe marginalization in order to coordinate a large number of services for severely marginalized adults. The “Cabina di regia” is a stable co-programming body, while the “Tavolo tecnico” deals with the study of specific situations. Nine actors of the third sector are present together with the representatives of the municipality. The coordinating function of this system is in the hands of the Municipality of Brescia. This type of permanent comparison allows the service system to develop joint projects between the municipal administration and the third sector organizations (20).

Indeed, these services provide people experiencing homelessness with food distribution points at the railway station, “street units” that go out during the night to visit those living on the streets, hot meals and shelter at social canteens, day-centers equipped with showers, washing machines and recreational spaces, up to the more classic dormitories and Housing First and Housing Led apartments—according to the latest intervention models—reintegration into work and accompaniment to specialized services.

In this cultural and social context, since 2018 the Association “Un Medico x Te” offers healthcare services to people experiencing homelessness in collaboration with some housing services (notably San Vincenzo de' Paoli and Congrega della Carità Apostolica).

It proposes innovative practices—compared to traditional public health services. Notably, it adopts a more unstructured approach, more centered on the actual needs of the patient experiencing homelessness and less on the figure of the doctor, especially aimed at the engagement of a typology of patients that often, for multiple reasons, escapes early from the care pathways—thus failing to exercise their right to health.

## 3 Key programmatic elements

### 3.1 First research phase—medical study

Many studies over the last decades proved that individuals experiencing homelessness demonstrate significant patterns of increased morbility and mortality in comparison to housed counterparts (21–23). In particular, since chronic liver diseases are very common among people experiencing homelessness (24), in order to investigate the presence of these pathologies, in 2023 the Association “Un Medico x Te” carried out a preliminary observational retrospective cohort study in the population experiencing homelessness who attended the housing services of Brescia.

#### 3.1.1 Materials and methods

This was a retrospective, observational, preliminary study. A total of 120 homeless subjects who regularly attend the collection centers of the Municipality of Brescia were included in the analysis. These individuals underwent periodic health checks as part of the standard healthcare services provided by the health authorities of the Province of Brescia.

In particular, the group comprised adult patients experiencing homelessness of both sexes who were attended by the Association during the period from 01/01/2023 to 01/12/2023. Participants were not selected through convenience sampling or randomization; rather, the cohort encompassed all individuals meeting the broad definition of the target population who accessed the Association's services within the specified timeframe. Indeed, unlike interventional trials, observational cohort studies do not employ pre-defined inclusion or exclusion criteria for subject selection but instead aim to capture a representative sample of the defined population to observe the natural history of exposures and outcomes as they occur in real-world settings (25, 26). This methodological approach ensures the preservation of real-world heterogeneity whilst providing insights into disease etiology and exposure-outcome associations that might be obscured in highly controlled experimental environments.

During these routine health checks, subjects were visited by a specialist doctor. After providing informed consent for their standard medical assessment, an abdominal ultrasound was performed to complete their health evaluation. The collected data, including the findings from the abdominal ultrasounds, were subsequently analyzed anonymously and retrospectively. No additional invasive procedures or interventions beyond the scope of routine clinical care were performed for the purpose of this study.

The presence of fibrosis and/or hepatic steatosis was assessed. Hepatic steatosis was quantified using the quantitative scale that provides degrees of steatosis: Grade 0 = absent, Grade 1 = medium, Grade 2 = moderate, Grade 3 = marked (24).

Various general information was also collected on this population such as age, alcohol consumption, duration of homelessness.

In house-developed software, powered by TWIN Informatic Solution (Castel Mella, Brescia, Italy) was used to collect the data.

The collected data were analyzed retrospectively and anonymously to identify the presence and characteristics of any evident liver problems. The data obtained from the homeless subjects will be compared with the data from the general population.

#### 3.1.2 Ethical considerations

This is a Retrospective Observational Study (ROS). ROS are considered non-interventional studies but involve the review of existing data. Indeed, in our study, the data were collected during a normal, pre-existing clinical check-up with patient's no risk and they were analyzed retrospectively and anonymously.

Informed consent was obtained from all subjects for their general medical assessment and the procedures performed as part of their routine care.

The anonymity of the data was ensured prior to analysis, thus protecting patient privacy and confidentiality. The study adheres to the ethical principles outlined in the Declaration of Helsinki (27) and the International Council for Harmonization of Technical Requirements for Pharmaceuticals for Human Use (ICH) Guideline E6 (R2) on Good Clinical Practice (28) and it does not require ethics committee approval, as recently pointed out (29).

#### 3.1.3 Results

Males were the clear majority compared to females (72.5 vs. 27.5%). The mean age of the subjects analyzed was 52.6 ± 4.7 years (mean + SD). The presence of hepatic steatosis was highlighted in 43.2% of patients, which represents a value more than double that of those highlighted in the general population. It is interesting to note that advanced degrees of steatosis (Grade II and III) were present in 42.3% of the total patients with steatosis, who stated in 76% of cases a consumption >2 glasses of wine and/or beer per day and denied the use of spirits. It is noteworthy that the degree of steatosis was related to the duration of the homeless condition (Grade 0 = 21.4 ± 2.4 months, Grade II = 37.3 ± 2.3 months, Grade III = 49.6 ± 2.6 months). In addition, to confirm the advanced stage of liver disease, 21.7% of subjects with Grade II–III presented cirrhotic lesions while only half of these report to be affected by hepatitis B and/or C. Notably, these values are respectively greater that 15 and 10 times the prevalence of those diseases in general population (30).

Concurrently, over time, and particularly throughout this research endeavor, the Association's medical professionals have developed evidence indicating that, considering the substantial population of homeless individuals residing in the city of Brescia, they are only able to engage with and provide care for a minority segment. This segment is comprised of those who are supported by housing services' social workers that monitor their journey and access to healthcare in a continuous and structured manner. Moreover, if there are numerous studies in the literature that investigate the “bad” health conditions of people experiencing homelessness, there is still a paucity of studies that delve into the experiences of homeless users when approaching health services (31, 32).

### 3.2 Second research phase—socio-educational study

Thus, to gain a better understanding of the physiognomy of the population experiencing homelessness in Brescia and to promote practices that are more responsive to the actual demand for health and care of the aforementioned population, in 2023 the Association set out to promote a study on more effective strategies to approach people experiencing homelessness and to provide them both preventive and therapeutic medicine.

Therefore, the Association asked the CESPEFI (Research Center on Family and Childhood Education of Catholic University of Sacred Heart) to conduct a socio-educational research project in order to investigate aspects of life and health, but also of social behaviors, which characterize the population experiencing homelessness within Brescia urban area.

The results of this study were then discussed and problematized by CESPEFI with volunteer clinicians in relation to their usual way of receiving and following up patients, in order to “adapt” the health services dedicated to the population experiencing homelessness in Brescia on their actual needs.

Consequently, in light of the new knowledges acquired through the socio-educational research design, they have experimented innovative approaches in their clinical practice and research—as we will better describe in the following paragraphs.

Specifically, the objectives of the second phase of the research are manifold and closely interconnected:

1) to engage with other practitioners who work with this homeless population in the city of Brescia (e.g., the “Menni” canteen, associations operating at the railway station, etc.), with a view to sharing approaches and practices for “reaching out” to those living in situations of extreme marginalization;
2) to gather novel clinical/epidemiological and “existential” data;
3) to experiment with interdisciplinary interpretative approaches (medicine and pedagogy);
4) to design relational and operational practices that enable the removal of “exclusion mechanisms” which effectively deprive a segment of potential service users of access to specific social protection and healthcare provisions.

#### 3.2.1 Material and methods

The first part of this research—of an exploratory nature—involved the implementation and analysis of focus groups conducted with healthcare practitioners (volunteers with the “Un Medico x Te” Association) and social workers (employees of organizations affiliated with the Severe Marginalization Panel of Brescia Municipality and local volunteers), in order to (i) identify the principal health-related items of greatest interest for the purposes of this research and (ii) evaluate the most effective strategies for approaching homeless individuals based on the experience of services specifically dedicated to this target.

Based on the evidence emerging from the first phase of the socio-educational study, and due to the specificity of the contextual elements and the population target, it was deemed appropriate to adopt an ethnographic methodological framework (33, 34), encompassing pure observation at very low-threshold services, participant observation at low/medium-threshold services, and finally interviews with service users accommodated in residential facilities (35–37).

Accordingly, from February up to August 2023 the following activities were undertaken:

- 1 preparatory meeting with the Adult Severe Marginalization Panel of Brescia Municipality
- 2 observations at breakfast distribution carried out by Sister Paola (Sisters Servants of Charity) and volunteers in the railway station forecourt in Brescia
- 1 participant observation on outreach with the City Angels Association
- 1 participant observation at L'Angolo Day Center
- 1 participant observation at the Help Center
- 1 participant observation at the Perlar Holiday Day Center
- 4 interviews with residents of San Vincenzo via Milano, Caritas Refuge, and Asilo Pampuri (one person with 1 year's experience of street life, two people with years of street life experience, one person who avoided street life for health reasons; one woman and three men; two under 40 years of age, two approaching 60), together with consultation with the respective service educators.

The exclusive effective eligibility criterion for participants was their presence in the designated places and services, indicated by the volunteers or professionals operating therein.

#### 3.2.2 Ethical considerations

Regarding the ethical oversight framework for the second research phase, all its activities adhere to rigorous educational ethnographic research methodologies. While no institutional Ethics Committee approval was required under institutional regulations of Università Cattolica del Sacro Cuore, the research underwent thorough legal validation at the competent university offices. The relevant documentation is retained within university authorities and is available for verification upon request.

In detail, focus groups with healthcare professionals were organized in collaboration with the Association Un Medico × Te. Participants—on a voluntary basis—were fully informed regarding the procedures and objectives of the focus group, as well as data handling and privacy measures. Data collected were anonymised, aggregated, and securely stored at the Research Center.

Similarly, for the focus group involving social operators, prior written notification was sent to the Municipality of Brescia and to all institutions active within the adult severe marginalization panel. Participation was voluntary. Explicit informed consent was obtained from attendees for video recording the sessions, which were subsequently transcribed and securely archived. The aggregated, anonymised results were presented back to participants in a plenary meeting to ensure transparency and allow verification of findings.

The phase of ethnographic fieldwork (pure observation) was conducted following classical ethnographic methodology. The supporting organizations were fully informed about the study aims and data handling, with strict adherence to preserving anonymity of both observed subjects and operators or volunteers. The researcher maintained a presence that was as unobtrusive and respectful as possible, fostering collaboration with operators and volunteers in the observed environments. Indeed, the researcher developed direct relationships with the operators and, through them, indirect relationships with the homeless population.

Data were recorded exclusively through detailed field notes that encapsulate the researcher's reflective observations rather than instantaneous “snapshots” of events. These notes respect the anonymity and dignity of all individuals encountered during the observational process.

With regard to interviews, a previous dialogue was held with staff at hosting facilities, who introduced the research to potential interviewees. Participation was entirely voluntary and based on informed consent, with explicit explanations provided about the study purpose, confidentiality, and data use. Interviews were video recorded and transcribed only with participants' consent. Recordings and transcripts are securely stored at the Research Center.

No coercion or incentives were offered to participants, and care was taken throughout to respect interviewees' autonomy, including their freedom to speak or remain silent. The interview process was designed to safeguard psychological and emotional wellbeing at all stages.

#### 3.2.3 Results

They were encountered considerably more men than women (ratio of 10:1 in a summary average), more foreign nationals than Italians (ratio of 6:4).

Their age ranged approximately from 18 to nearly 80 years (with significant distribution from approximately 40 to 60 years).

The numbers encountered varied from 120 people at the railway-station food distribution, to 80/100 with City Angels, similar amounts for L'Angolo Day Center when operating at full capacity, down to 30/40 at Perlar's (Saturday and Sunday) Day Center.

The notes from fieldwork, observation and participant observation activities, as well as the recording and transcription of semi-structured interviews, were subsequently subjected to content analysis.

Specifically, the content analysis led to the construction of codes, namely meaning units, and consequently to the definition of code families or code aggregations, according to a criterion of describing processes and activities. In particular, the following results were identified: sliding mechanisms and exclusion dynamics; “impactful” elements, namely those whose significant recurrence determines a considerable impact on the living conditions of homeless people; mechanisms for exiting street life and elements of personal resilience; hierarchy of priorities of homeless people; outreach practices.

##### 3.2.3.1 “Sliding” mechanisms and exclusion dynamics

The research underscores some key events that trigger the “sliding” mechanisms or dynamics of progressive social exclusion among individuals experiencing homelessness. These triggers can be divided into personal factors and environmental factors

The personal factors that have been identified are as follows.

Illness, bad health conditions that prevent individuals from working or even managing personal care can lead to temporary or permanent loss of autonomy. Without timely and adequate support, this often results in cascading consequences such as job loss, deterioration of relationships, and eventual experiencing homelessness.

Furthermore, any form of addiction profoundly affects the priorities of individuals experiencing homelessness. According to both persons experiencing homelessness and service providers, addiction dictates their focus, making all aspects of life subordinate to satisfying this need.

As for more environmental factors, they recur as follows.

Challenging family situations, relational poverty within families exacerbate material poverty. The lack of functional support from a primary family network often accelerates social exclusion.

Both individuals experiencing homelessness and service providers report that untimely interventions by social services contribute to worsening exclusionary dynamics, pushing individuals further into experiencing homelessness. “*But you can't think that you can live my whole life like this, day after day [...] Oh breakfast, where shall we have breakfast? because you have to eat, where shall we have lunch and where shall we also have dinner?*

*[...] I waited two months for a place to sleep at night, again a dormitory where I was the first time.”*

A job loss, and the inability to secure a new job quickly after losing one, depletes financial resources, often leading to debt. This creates a “point of no return,” marked by the breakdown of relationships, loss of stable housing, and eventual experiencing homelessness.

Bureaucratic complexity, that is excessively complicated administrative processes, is described as “social violence” (38, 39). These mechanisms create impasses or unsustainable conditions, exacerbating marginalization. Examples include issues related to obtaining residency permits, health cards, or other essential documentation.

These results confirm at the local level the findings present in the relevant scientific literature both at national and international levels (3, 17, 40).

##### 3.2.3.2 Key findings on the living conditions of people experiencing homelessness

Several critical factors impact the living conditions of individuals experiencing homelessness. These factors fall into two broad categories: personal challenges and environmental complexities.

Personal challenges include, first, an impaired communication: a significant proportion of people experiencing homelessness exhibit compromised communication skills, stemming from factors such as limited Italian proficiency, potential cognitive decline (possibly linked to substance dependence), or reported psychiatric conditions. This inhibits effective interaction, causing frustration, shame, and social withdrawal in individuals experiencing homelessness, while demanding exceptional communication skills from social workers.

Moreover, all interviewed participants had a history of substance and/or alcohol dependence. Social workers agree that active addiction is a pervasive issue, dominating nearly every aspect of life of people experiencing homelessness and hindering their escape from margination.

The study noted the presence of unaccompanied foreign nationals reaching adulthood, who can become particularly vulnerable. Similarly, the situation of women, particularly foreign nationals, often under the influence of drugs and seemingly controlled by men, is a cause for concern. These women appear fearful and resigned, suggesting a state of profound crisis.

Furthermore, social and health workers in recent years have observed a rise in population experiencing homelessness over the age of 65, creating new demands on social and healthcare services. Existing provisions for older adult are often inadequate to address the complex needs of this population, while shelters for people experiencing homelessness are not equipped to provide geriatric care.

The environmental factors that have been identified are as follows.

People experiencing homelessness often face a confluence of complex and specific issues, defying easy categorization. This complexity hinders problem identification and appropriate intervention, leading to a sense of malaise and disengagement among both individuals experiencing homelessness and social workers.

Another growing problem is the inadequate placement of individuals experiencing homelessness being discharged from hospitals. The lack of formalized institutional pathways results in patients experiencing homelessness being placed in shelters ill-equipped to handle their complex health (including psychiatric) needs, often for extended periods. This phenomenon highlights the urgent need for interdisciplinary collaboration between social, healthcare, and mental health services.

Both individuals experiencing long-term homelessness and those with shorter histories of poverty benefit from long-term, continuous support. Relapse into homelessness is often triggered by isolation following adverse life events. Consistent outreach and support, including domiciliary assistance, could mitigate these relapses.

The last result pertains the highly skilled workforce. Social workers generally possess education/pedagogy training; however, their role requires performing functions related to other professions. They have reported that additional knowledge is constructed within the field, as well as consulting with other's experience to reinforce acquired knowledge and practices.

“*We aim to give the most complete and humane answers to the people we meet. These people have incredible stories, and we are fully committed to them.”*

##### 3.2.3.3 Mechanisms of homelessness exit

This current study outlines key mechanisms facilitating the transition out of homelessness, categorized as either personal or environmental factors (41).

The following individual attributes and behaviors are identified as contributing to successful exits from experiencing homelessness: self-determination, in terms of a significant level of personal motivation; adaptive coping strategies, such as the employment of strategies to maintain engagement and purpose (e.g., seeking activities to occupy time “*I‘ve always thought that if I keep busy, I don't think about doing anything else.”*); trust and reliance on support staff, that is the capacity to develop trusting relationships with social and health workers; addiction cessation, in terms of total freedom from substance dependencies; relationship care, that means actively nurturing interpersonal relationships and affective bonds *(“I had my son as well, who was quite young, and I used to bring him to the community. That motivated me to get involved, because if you are not motivated, you can't move forward. You end up being what you are, because when you're on the street, you have nothing to do and nothing to lose.”*); preservation of functional skills, because the retention of residual skills enables a degree of independent living, facilitating social interaction and the establishment of a more autonomous daily routine.

Also the following external factors related to support services and their delivery are identified as contributing to successful exits from homelessness.

First of all, the proactive engagement by social services—that is outreach-oriented service delivery, characterized by proactive engagement with individuals experiencing homelessness—involves actively seeking out individuals in need, particularly in locations where support access is most challenging. “*Because I saw that they were people who always did something for me. So, I relied on them.”*

Service responsiveness, in terms of timeliness and efficiency of service provision, particularly at the initial intake stage, helps to find better and early solutions for exclusion dynamics. Delays and inefficiencies can erode trust and undermine engagement, leading to disaffection and a loss of confidence in available support.

Extended periods of in-home support following interventions such as addiction treatment are consistently identified as a crucial factor for sustainable social integration. The absence of such ongoing assistance can negate the long-term benefits of even extensive and costly therapeutic interventions. Consistent domiciliary support, particularly during periods of personal crisis, is deemed essential for maintaining a baseline level of independent living.

##### 3.2.3.4 Health in the priority scale of people experiencing homelessness

Among the priorities of people experiencing homelessness, it is perceived that health is not an “everyday” issue: it only becomes relevant when life is at risk.

“*When you're out and about, you don't really think about your health as much, especially if you're just trying to get high. I don't really think about it anymore. A few days later and the problem's still there. It's not going anywhere! And that's done. What happens to you happens. But if you don't realize it yourself, you'll never know how to get there.”*

It is also recognized that most people experiencing homelessness do not have a culture of health care or prevention.

But the value of health care is not absent: in situations of access to medical care, it is given a positive value of effectiveness. Moreover, health care is often seen as the key to social care. Therefore, the issue of health care can be seen as a kind of “strategic asset” in the experience of homelessness.

These findings are significantly corroborated by similar research conducted both within Italy and internationally (18, 31).

##### 3.2.3.5 Effective and ineffective practices in approaching individuals experiencing homelessness

Based on the present investigation, certain elements, processes, and relational/communicative modalities appear to enhance the efficacy and functionality of engaging with individuals experiencing homelessness, such as:

- spatial and temporal proximity, in terms of ensuring easy access to support services precisely where and when individuals experience immediate need;
- enhanced communication skills—employing proficient communication strategies by support staff compensate for expressive difficulties in individuals experiencing homelessness, fostering a sense of being heard and understood;
- provision of explicit guidance and concrete support mechanisms;
- continuity of support workers—maintaining consistent contact with the same support workers is perceived as a prerequisite for fostering trust and enabling individuals experiencing homelessness to engage with more structured care pathways. Participants consistently referenced specific support workers by name, highlighting the importance of trusted individuals in facilitating positive change.

On the other side, the following factors are identified as hindering successful engagement:

- excessive bureaucracy and rigid protocols—inflexible rules and overly complex procedures are difficult and unsustainable within the lived realities of individuals experiencing homelessness;
- impersonal, service-oriented relationships, in terms of prioritizing transactional service delivery over building rapport and trust. Factors such as high staff turnover and impersonal communication styles that disregard the circumstances of individuals experiencing homelessness represent significant barriers to engagement. Such practices can reinforce a sense of exclusion, deterring the development of trusting relationships.

Similar results have been confirmed by several recent international studies, particularly with regard to assertive outreach models designed to bring services directly to individuals within the community (rather than relying on individuals to self-present to services); but also in relation to the provision of “wraparound” health and social services (16–18).

##### 3.2.3.6 Development of best practice guidelines

As a result of this study, volunteer physicians from “Un Medico x Te,” in collaboration with CESPEFI researchers, following a maieutic approach (42), developed a set of guidelines aimed at informing their clinical practice. These guidelines emphasize, on one hand, prioritizing the patient-practitioner relationship. This means ensuring consistent, welcoming interaction, maintaining clear and concrete communication, and employing attentive and respectful listening skills.

On the other hand, they guarantee care pathway sustainability, by focusing on sustained accompaniment and support, facilitating access to resources, and promoting effective treatment adherence.

## 4 Discussion

Analysis of data collected from people experiencing homelessness, support workers, volunteers, and institutional representatives has generated the following key findings regarding healthcare provision for vulnerable population experiencing homelessness.

First of all, it has been shown that initial engagement with healthcare services often acts as a gateway to more comprehensive and structured support from social services. While awareness of this pathway is growing, further development of functional processes is essential. A specific area for improvement is the coordination of hospital discharge for individuals experiencing homelessness who require immediate housing for convalescence.

Secondly, the provision of care to individuals experiencing homelessness necessitates specific methodologies, timeframes, and resources, in order to reach this population, to insure them adequate health-care and consequently to avoid perpetuating further exclusion. Specifically, appropriate medical care for this target requires a multidisciplinary and integrated approach (43, 44).

Consequently, clinical practice is enhanced through the incorporation of insights, techniques, and communicative strategies from allied disciplines such as education sciences and psychology—as it has already been shown by significant studies in sociology and education (43). This integration addresses communication and relational vulnerabilities prevalent among patients experiencing homelessness.

Furthermore, effective integration and coordination between various services and operators involved in the care of individuals experiencing homelessness is fundamental. This facilitates the reconstruction of fragmented medical histories, ensures optimized organization of appointments, therapies, and treatments, promotes economic sustainability of care pathways, and enables holistic care addressing the interconnected housing, legal, economic, health, and social dimensions of a patient's life.

And, ultimately, the adoption of internal guidelines has influenced both the clinical approach and research methodology employed by the physicians of the Association “Un Medico x Te.” This has included moving beyond traditional clinic-based care to conduct outreach in locations frequented by experiencing homelessness individuals (e.g., food distribution points), leveraging the expertise of operators with established relationships with the population experiencing homelessness (e.g., social workers, community nurses, volunteers), and utilizing efficient data collection methods to minimize interference into the lives of people experiencing homelessness.

## 5 Acknowledgment of any conceptual or methodological constraints

This study acknowledges several conceptual and methodological limitations that warrant consideration.

The adoption of a multi-perspective and multi-disciplinary approach, while valuable, often contrasts with the specialized training of practitioners. Traditional training frameworks, deeply rooted in individual disciplines, may lack sufficient emphasis on the significance of relational dynamics and sustained accompaniment in effective care provision.

As a voluntary medical assistance organization, “Un Medico x Te” faces inherent limitations imposed by the bureaucratic structures of the Italian National Health Service, to which it is nonetheless accountable.

The precarious living circumstances of experiencing homelessness individuals, particularly those lacking regular housing support, can substantially compromise the continuity and, therefore, the efficacy of care pathways.

The heterogeneity of the population experiencing homelessness in Brescia (e.g., older adults with substance addiction, unaccompanied foreign minors, those with mental health conditions) presents challenges in systematically analyzing data derived from this medical case study. Subgroup-specific analyses, pathways, and interventions may be more appropriate.

It is important to acknowledge that this report documents a preliminary research attempt. Further investigations are required to develop and evaluate innovative strategies for: 1) effectively engaging with experiencing homelessness patients, and 2) implementing appropriate medical assistance protocols.

To validate the approach to individuals experiencing homelessness outlined in the guidelines presented herein, and to enhance understanding of their health status, “Un Medico x Te” is currently organizing an observational cohort study. This study will investigate the prevalence of cardiovascular risk factors, and chronic pulmonary, hepatic, and renal diseases within the population experiencing homelessness of the province of Brescia.

## 6 Conclusion: catalyzing community-based systemic change

This community case study has stimulated reflection on the processes involved in healthcare provision for people experiencing homelessness within both social services and the local medical community, fostering openness to systemic and integrated community dynamic revision and generation.

Thus, this experience of reflexive practice (45) represents a form of responsibility, as it stimulates attention to the social consequences of professional knowledges and practices, activating democratic attitudes and behaviors and seeking to generate inedited solutions to potential conflictual social dynamics. In this perspective, reflexive practice allows exploring “worlds of possibilities” and activating a broadening of perspectives, awarenesses and social and health interventions (46) in order to improve health care policies and better grant the right to healthcare for all the Community.

## Data Availability

The raw data supporting the conclusions of this article will be made available by the authors, without undue reservation.

## References

[1] Pleace N. Researching homelessness in Europe: theoretical perspectives. Eur J Homelessness. (2016) 10:19–44. Available online at: https://eprints.whiterose.ac.uk/111071/

[2] Fitzpatrick S. Explaining homelessness: a critical realist perspective. Hous Theory Soc. (2005) 22:1–7. 10.1080/14036090510034563

[3] Fitzpatrick S. Homelessness: causation. In:Smith SJ, editor. International Encyclopedia of Housing and Home. Oxford: Elsevier (2012). p. 15–24. 10.1016/B978-0-08-047163-1.00347-7

[4] Batterham D. Defining “at-risk of experiencing homelessness”: re-connecting causes, mechanisms and risk. Hous Theory Soc. (2017) 36:1–24. 10.1080/14036096.2017.1408678

[5] Consoli T Meo A editors. Homelessness in Italia. Biografie, Territori, Politiche. Milano: FrancoAngeli (2020).

[6] Aubry T Bloch G Brcic V Saad A Magwood O Abdalla T . Effectiveness of permanent supportive housing and income assistance interventions for homeless individuals in high-income countries: a systematic review. Lancet Public Health. (2020) 5:e342–80. 10.1016/S2468-2667(20)30055-4 32504587

[7] Tsemberis S. Housing First: ending homelessness, promoting recovery, and reducing costs. In:O'Flaherty B Ellen I, editors. How to House the Homeless. New York: Russell Sage Foundation (2010). p. 37–56.

[8] Padgett D Henwood B Tsemberis S. Housing First: Ending Homelessness, Transforming Systems, and Changing Lives. New York: Oxford University Press (2016). 10.1093/acprof:oso/9780199989805.001.0001 27903173

[9] Marpsat M. The problem of definitions: points of similarity and difference. In: Presented at: CUHP Thematic Network Conference. Bruxelles (2005).

[10] OECD. Indicator HM1.1. Housing stock and construction. Affordable Housing Database (2024). Available online at: https://oe.cd/ahd (Accessed June 16, 2025).

[11] UN UN Human Settlements Programme - Open-ended Intergovernmental Expert Working Group on Adequate Housing for All First session Nairobi 9–11 9–11 December 2024 Item 3 of the provisional agenda State of efforts to progressively realize adequate housing for all (2024). Available online at: https://www.developmentaid.org/api/frontend/cms/file/2023/12/2416772e.pdf (Accessed July 28, 2025).

[12] UN General Assembly. Inclusive Policies and Programmes to Address Homelessness. New York: United Nations (2023).

[13] Westbrook M Robinson T. Unhealthy by design: health and safety consequences of the criminalization of homelessness. J Soc Distress Homelessness. (2021) 30:107–15. 10.1080/10530789.2020.1763573

[14] Bensken WP Krieger NI Berg KA Einstadter D Dalton JE Perzynski AT. Health status and chronic disease burden of the homeless population: an analysis of two decades of multi-institutional electronic medical records. J Health Care Poor Underserved. (2021) 32:1619–34. 10.1353/hpu.2021.0153 34421052 PMC8477616

[15] Wickham S. Effective interventions for experiencing homelessness populations: the evidence remains unclear. Lancet Public Health. (2020) 5:e304–5. 10.1016/S2468-2667(20)30120-1 32504583

[16] D'Souza MS Mirza NA. Towards equitable health care access: community participatory research exploring unmet health care needs of homeless individuals. Can J Nurs Res. (2022) 54:451–63. 10.1177/08445621211032136 34387510 PMC9605994

[17] Clifford B Wood L Vallesi S Macfarlane S Currie J Haigh F . Integrating healthcare services for people experiencing homelessness in Australia: key issues and research principles. Integr Healthc J. (2022) 4:e000065. 10.1136/ihj-2020-000065 37440845 PMC10241025

[18] Kiser T Hulton L. Addressing health care needs in the homeless population: a new approach using participatory action research. Sage Open. (2018) 8. 10.1177/2158244018789750

[19] Comune di Brescia. Popolazione residente per genere (2024). Available online at: http://dati.comune.brescia.it/dataset/popolazione-residente-per-genere-01-01-2024 (Accessed June 16, 2025).

[20] Comune di Brescia Ambito 1 Brescia e Collebeato. Piano di zona 2021–2023 (2022). Available online at: https://www.comune.brescia.it/lfs/servizi/servizisociali/TraspDebInformativo/Documents/PIANO%20DI%20ZONA%202021-2023%20AMBITO%201-BRESCIA%20-%20VERSIONE%208%20FEBBRAIO%202022.pdf#:~:text=Il%20Piano%20di%20Zona%20%C3%A8%20lo%20strumento,rafforzamento%20dei%20servizi%20e%20la%20loro%20innova%2D (Accessed June 16, 2025).

[21] Richard L Carter B Wu L Hwang SW. Disparities in all-cause mortality among people experiencing homelessness in Toronto, Canada during the COVID-19 pandemic: a cohort study. Front Public Health. (2024) 12:1401662. 10.3389/fpubh.2024.1401662 39185124 PMC11341496

[22] Chang JS Saxton K Bright G Jorden MA Gutierrez A Xia K. Deaths of profound despair: a retrospective cohort study of mortality among people experiencing homelessness. PLoS ONE. (2023) 18:e0281912. 10.1371/journal.pone.0281912 36795773 PMC9934450

[23] Baggett TP Hwang SW O'Connell JJ Porneala BC Stringfellow EJ Orav EJ . Mortality among homeless adults in Boston. JAMA Intern Med. (2013) 173:189–95. 10.1001/jamainternmed.2013.1604 23318302 PMC3713619

[24] Gerstenmaier J Gibson R. Ultrasound in chronic liver disease. Insights Imaging. (2014) 5:441–55. 10.1007/s13244-014-0336-2 24859758 PMC4141343

[25] Hennekens CH Buring JE. Epidemiology in Medicine. Boston, MA; Toronto, ON: Little, Brown and Company (1987).

[26] Rothman KJ Greenland S. Modern Epidemiology. Philadelphia, PA: Lippincott Williams & Wilkins (2008).

[27] World Medical Association. World medical association declaration of Helsinki: ethical principles for medical research involving human subjects. JAMA. (2013) 310:2191–4. 10.1001/jama.2013.281053 24141714

[28] International Council for Harmonisation of Technical Requirements for Pharmaceuticals for Human Use (ICH). ICH Harmonised Guideline (1996). Available online at: https://www.ich.org (Accessed June 16, 2025).

[29] Pasini E Corsetti G Romano C Aquilani R Scarabelli T Chen-Scarabelli C . Management of anaemia of chronic disease: beyond iron-only supplementation. Nutrients. (2021) 13:237. 10.3390/nu13010237 33467658 PMC7830481

[30] Cheemerla S Balakrishnan M. Global epidemiology of chronic liver disease. Clin Liver Dis. (2021) 17:365–70. 10.1002/cld.1061 34136143 PMC8177826

[31] Callegarin S Camuccio C Cutrone F. Salute e malattia AI margini della società: uno studio qualitativo sui Senza Dimora. J Health Care Educ Pract. (2020) 2:55–64. 10.14658/pupj-jhcep-2020-2-5

[32] Nickasch B. Healthcare experiences of the homeless. J Am Assoc Nurse Pract. (2009) 21:39. 10.1111/j.1745-7599.2008.00371.x 19125894

[33] Bove C. Il metodo etnografico. In:Mortari L Ghirotto L, editors. Metodi Per la Ricerca Educativa. Roma: Carocci (2019). p. 101–42.

[34] Mortari L. Cultura Della Ricerca E Pedagogia. Roma: Carocci (2007).

[35] Gobo G. Descrivere il mondo. Teoria e pratica del mondo etnografico in sociologia. Roma: Carocci (2001).

[36] Silverman D. Doing Qualitative Research. London: SAGE (2000).

[37] Atkinson P Coffey A Delamont S Lofland J Lofland L., editors. Handbook of Ethnography. London: SAGE (2001). 10.4135/9781848608337

[38] Porcellana V. Diventare “senza dimora”. Politiche e pratiche del welfare alla lente dell'etnografia. Antropologia. (2018) 5:113–32. 10.14672/ada20181390113-132

[39] Wacquant L. Punire I Poveri: Il Nuovo Governo Dell'insicurezza Sociale. Roma: DeriveApprodi (2006).

[40] Parsell C. Experiencing Homelessness: A Critical Introduction. Cambridge: Polity Press (2023).

[41] Hill D McPhee B Lazanja S. From roots to home: visualizing the journey from experiencing homelessness to housing. Int J Homelessness. (2025) 5:1–8. 10.5206/ijoh.2023.3.2226339985557

[42] Dolci D. La Struttura Maieutica E L'evolverci. Scandicci: La Nuova Italia (1996).

[43] Gnocchi R editor. Homelessness e Dialogo Interdisciplinare. Analisi e Confronto tra Modelli Diversi. Roma: Carocci (2009).

[44] McDonald J Hale K Kirkwood T. Excellence in homelessness services: evidence-based frontline practices. Int J Homelessness. (2024) 4:139–57. 10.5206/ijoh.2023.3.1665539985557

[45] Amadini M. Reflexivity. In:Colombo M Gilardoni G, editors. Intercultural Issues and Concepts. A Multi-Disciplinary Glossary. Bruxelles: Peter Lang (2021). p. 185–98.

[46] Amadini M Pasini A. How can educational research assume an intersectional approach? A case study on experiencing homelessness in Brescia. In:Scuola Democratica, editor. Proceedings of the Third International Conference of the journal Scuola Democratica. Education and/for Social Justice. Vol. 1: Inequality, Inclusion, and Governance. Associazione “Per Scuola Democratica”. Rome (2025). p. 85–92.

